# Overview of Optical Biosensors for Early Cancer Detection: Fundamentals, Applications and Future Perspectives

**DOI:** 10.3390/biology12020232

**Published:** 2023-02-01

**Authors:** Mohammad Y. Azab, Mohamed Farhat O. Hameed, Salah S. A. Obayya

**Affiliations:** 1 Mathematics and Engineering Physics Department, Faculty of Engineering, University of Mansoura, Mansoura 35516, Egypt; 2 Center for Photonics and Smart Materials, Zewail City of Science and Technology, October Gardens, 6th of October City, Giza 12578, Egypt; 3 Department of Electronics and Communications Engineering, Faculty of Engineering, University of Mansoura, Mansoura 35516, Egypt

**Keywords:** early cancer detection, optical biosensors, plasmonic biosensor, label free, metamaterial

## Abstract

**Simple Summary:**

Due to the increase in cancer incidence worldwide, a necessity for early cancer detection arises. Since the conventional detection and treatment techniques are costly, great efforts have been exerted in developing new methods for early cancer detection. Highly reliable and efficient methods are based on optical detection techniques, which are sensitive, accurate, rapid, noninvasive and immune to external fields. This includes optical biosensors based on plasmonic waveguides and metamaterial. Additionally, many optical detection methods have been adopted using the colorimetric technique, optical coherence tomography, surface-enhanced Raman spectroscopy and reflectometric interference spectroscopy. This review paper introduces the basic features of each optical detection technique focusing on recent research works toward cancer detection.

**Abstract:**

Conventional cancer detection and treatment methodologies are based on surgical, chemical and radiational processes, which are expensive, time consuming and painful. Therefore, great interest has been directed toward developing sensitive, inexpensive and rapid techniques for early cancer detection. Optical biosensors have advantages in terms of high sensitivity and being label free with a compact size. In this review paper, the state of the art of optical biosensors for early cancer detection is presented in detail. The basic idea, sensitivity analysis, advantages and limitations of the optical biosensors are discussed. This includes optical biosensors based on plasmonic waveguides, photonic crystal fibers, slot waveguides and metamaterials. Further, the traditional optical methods, such as the colorimetric technique, optical coherence tomography, surface-enhanced Raman spectroscopy and reflectometric interference spectroscopy, are addressed.

## 1. Introduction

Due to the increasing rate of cancer incidence around the globe, a high necessity for early detection and diagnosis has been raised. Statistics show that the rate of new cases is 442.4, with a death rate of 158.3 per 100,000 cases per year [1]. Cancer is a genetic disease, which can be developed in the human body because of an error in cell division, DNA damage by harmful substances and parental inheritance [2]. Cancer can be referred to as an abnormal clone of cells characterized by uncontrollable growth rate [2]. In addition, unlike normal cells, the cancerous cells’ division can spread beyond the neighboring cells and cause tumors. In this regard, two types of tumors may appear in the living body, i.e., benign and malignant tumors [3]. Malignant tumors present cells that grow uncontrollably and spread locally and/or to distant sites [3]. A malignant tumor can invade other sites and is considered cancerous. Malignant tumors use the lymphatic system or bloodstream to spread throughout the body, which is called metastasis [3]. Metastasis can occur anywhere in the body and is usually found in the liver, lungs, brain and bone. On the other hand, benign tumors can stay in their primary location without invading other sites in the body. They do not spread to local structures or to distant parts of the body. Benign tumors tend to grow slowly and have distinct borders. In addition, benign tumors do not grow again after surgical removal, which differs from cancer tumors [3]. Additionally, blood cancer does not include tumors, but it has an uncontrollable growth of blood cells, which upsets the blood balance. Unfortunately, cancer incidence cannot usually be realized until very late stages, which require painful and costly chemical, radiational or surgical treatment. Early malignancies are often missed due to small or sessile lesions, which are difficult to detect. Further, in late stages, it is possible to diagnose cancer incidence by traditional methods, namely lab tests, image tests and biopsy [4]. The lab test includes analyzing specific ingredients of the human body, such as urine or blood, and searching for cancer markers. Image tests, such as CT [5], MRI [6] and ultrasound [7], can obtain detailed images of the inside organs where tumors can be an indication of cancer. In biopsy, a human tissue of the desired organ is taken, and the pathologist looks at the tissue under a microscope and decides whether the tissue is cancerous or not [4]. The pathologist may need to run other tests to see if the tissue has cancer cells or not. As stated before, most of the conventional diagnosing techniques are applicable only in late stages where the cancer tumors have already emerged. Further, the biopsy technique can be applied in early stages, but this is dependent on the pathologist’s experience and decision [4].

Another promising method for diagnosis is to deploy biosensors to detect the incidence. Biosensors are characterized by their ease of use, high performance, low response time, and they overcome the human error [8]. It is possible to categorize biosensors into two categories: biorecognition based and transduction based [8]. Biorecognition-based biosensors rely on the biological interaction between the test sample and the bioreceptor, where the interaction can be catalytic or affinity based. On the other hand, transduction-based biosensors rely on the dependency of a specific physical property on the change in the sample properties. These physical properties can be electrochemical, optical, piezoelectric or thermal.

**Figure 1:**
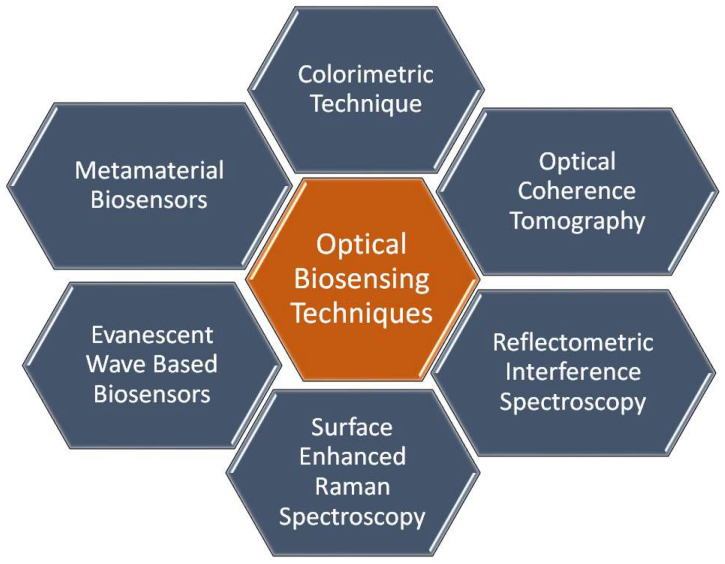
Different types of optical sensing mechanisms.

Optical biosensors are unique in their compact size, high sensitivity and immunity to stray fields [9]. It is possible to distinguish cancerous cells from normal cells via optical biosensors based on the difference in the refractive index. Cancer cells suffer from uncontrollable growth rate, where the protein content in a cancer cell is far larger than that in a normal cell. This causes a remarkable difference in the refractive index between cancerous and normal cells. Accordingly, the interaction between an incident electromagnetic wave and a normal cell will differ from the interaction with a cancer cell. Therefore, it will be possible to distinguish normal cells from cancer cells. To construct an optical biosensor to sense the change in the refractive index, a receptor is required to interact with the sample under study [9]. In this regard, various optical techniques, as shown in Figure 1, have been reported and studied, including evanescent-wave-based fluorescence [10], surface-enhanced Raman spectroscopy [11], reflectometric interference spectroscopy [12], colorimetric detection [13,14], optical coherence tomography [15], surface plasmon resonance [16], slot waveguides [17] and metamaterials [18]. In the following sections, a detailed review of each technique will be presented to highlight the recent research works on each one, as well as their advantages and disadvantages.

## 2. Optical Biosensing Techniques

### 2.1. Colorimetric Detection

**Figure 2:**
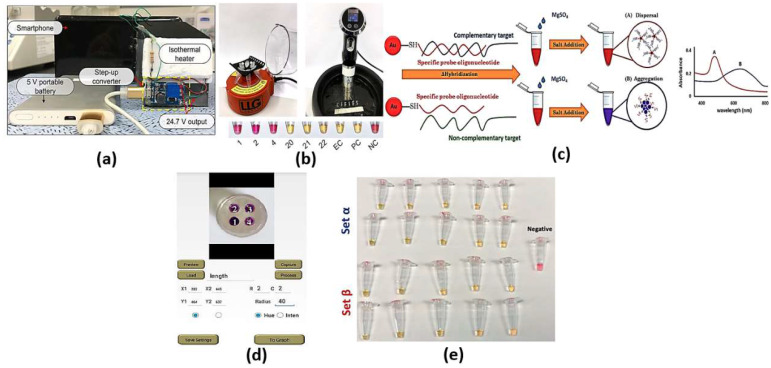
(**a**) Front side and back side of the i-Genbox LAMP box [21], (**b**) Left—mini-centrifuge used for DNA precipitation and preparation of the LAMP reaction mix; right—sous-vide stick heating water to different temperatures in a rubber ice bucket; bottom—results of 6 samples to test the simple setup relying on visual readout [22], (**c**) A schematic illustration of LAMP-GNP/DNA probe assay [23], (**d**) The interface of the smartphone app for hue value quantitative measurement [20] and (**e**) LAMP primer sets α and β, which can enable the amplification of synthetic samples of SARS-CoV-2 nucleic acids in a wide range of template concentrations [24].

Colorimetric cancer detection by nanomaterials is one of the promising techniques based on the absorption of exact colors upon incidence of optical signal on the test subject. The detection can be achieved using bare eye, simple camera or intensity-based optical detectors. Various bioassays have been adopted to identify and detect the sample to be scanned for color changes, such as loop-mediated isothermal amplification (LAMP), nanoparticles and silicon-nitride thin film. The LAMP bioassay is based on the amplification of DNA using DNA polymerase up to $10^{39}$ times. To detect the color change using bare eye via LAMP, one can use SYBR Green 1, hydroxynaphthol blue (HNB) or propidium iodide. In this regard, the color changes from orange to green for the SYBR Green I solution, while the HNB solution stays blue for positive reactions. However, the color becomes purple for negative reactions [19]. On the other hand, propidium iodide turns orange in negative reactions, while it is pink in positive reactions [19]. The high sensitivity of the LAMP assay may sometimes lead to false positive results due to cross-contamination or the samples’ carry-over during the experiment [19]. Cross-contamination can occur during the cis and trans priming among the oligonucleotide primers, where LAMP amplicon detection may be non-specific [19]. Such non-specific binding can be minimized through alternatively binding a quenching probe competition assay, real-time LAMP detection by turbidity and fluorescence resonance energy transfer [19]. Further, the need to design specific primers to target specific genes in the virus may require much more time for each set of primers for a specific virus to be designed and synthesized. In this regard, a pathogen detector based on hue quantitative molecular detection platform has been reported [20]. In addition, the reported smartphone-based platform colorimetric sensor shown in Figure 2a [21] based on integrated gene box (i-Genbox) with a LAMP chip has seven reaction chambers, which are capable of predicting the copy number of nucleic acids in the test samples. Moreover, the method presented in Ref [22] utilizes Phenol red as a pH-sensitive readout, relying on a distinct color change from pink to yellow in case of a positive reaction. The LAMP bioassay [22] reactions with different primers were compared via the specified equipment presented in Figure 2b, and a newly designed set targeting the gene encoding the tegument protein V67 provided the best results, enabling readout within 8−30 min. Further, the LAMP-based colorimetric method shown in Figure 2c for the detection of monocytogenes has been demonstrated, whereby the non-complementary target DNA sequence turns from red to purple, while the complementary target DNA remains red [23]. The platform, whose interface is shown in Figure 2d [20], combines smartphone-based real-time color analysis and colorimetric LAMP assay. Such combination [20] has successfully detected human immunodeficiency virus (HIV) in a plasma sample and human papillomavirus (HPV) in saliva samples/clinical vaginal swab samples. Further, the detection and amplification of severe acute respiratory syndrome coronavirus disease 2 (SARS-CoV-2) genetic sequences have been reported based on the LAMP method via in-house-designed initiators, as may be seen in Figure 2e [24]. Moreover, a wax-printed paper substrate forming a platform for a LAMP-based colorimetric sensor for dsDNA quantification has been reported and studied [25].

On the other hand, nanoparticles can be used as colorimetric probes for the detection of bio changes accompanied by the color changes. Bare eye detection is possible through gold nanoparticle probes, which are surrounded by the target sample in the form of RNA or DNA. Due to the plasmonic absorption of the dispersed AuNPs, the nanoparticles will be dispersed as a red solution even with added salt. However, the salt will cause the AuNPs to aggregate when the coating of the dsDNA or the virus around the AuNPs is missing, and a shift in the absorption peak will occur, which will change the solution color from red to purple.

Additionally, the change in color can be detected via observing the absorption spectrum for the desired sample. Based on localized surface plasmons, nanoparticles of dimensions that are much smaller than the incident wavelength can achieve high absorption. The absorption peak is dependent on the incident wavelength and the refractive index of the surrounding medium. Accordingly, the sample to be tested can be put on a substrate layer, which contains the nanoparticles, where the resonance absorption peak depends on the refractive index of the sample. In this context, a colorimetric biosensor based on highly catalytically active Au nanoparticle-decorated Bi_2_Se_3_ (Au/Bi_2_Se_3_) nanosheets has been proposed [26].

**Figure 3:**
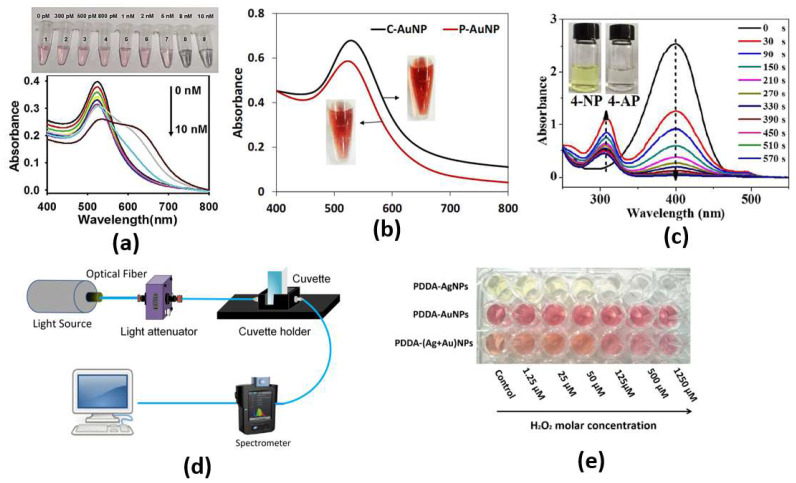
(**a**) Photographs of color change and typical UV–vis spectra upon addition of different concentrations of miR-21 [30], (**b**) UV−Vis absorption spectra of C-AuNPs and P-AuNPs. Inset: colors of AuNPs solution [27], (**c**) UV-vis absorption spectra of the reduction in 4-NP by NaBH4 in the presence of Au/Bi2Se3 nanosheets, with inserted photograph showing the color change of reduction in 4-NP [26], (**d**) The optical-fiber-based LSPR [29], (**e**) Evolution of colorimetric changes of PDDA-AgNPs, PDDA-AuNPs and PDDA-(Ag+Au)NPs [31].

Through both the color change from red to purple due to a conformational change of aptamer in the presence of SEB and the phenomenon of salt-induced AuNPs aggregation, monitoring can be achieved by naked eye or UV–vis spectrometer, as shown in Figure 3c, with a limit of detection as low as 160 pg/mL for the cancer biomarker [26]. Furthermore, an ultrasensitive microRNA-155 (miR-155) optical biosensor for breast cancer early diagnosis has been developed [27]. The proposed cancer biosensor is capable of detection of very low concentrations of miR-155, as shown in Figure 3b, where a detection limit of 100 aM has been reported [27]. In addition, a paper-based colorimetric immunoassay method for sensitive detection of a pancreatic cancer biomarker with a limit of detection of ∼10.0 ng m$L^{-1}$ has been reported [28]. The reported paper-based biosensor has an enhanced detection sensitivity, which is 10-fold that of the AuNP-based colorimetric immunoassay without signal amplification [28]. Further, Chang et al. have reported a fiber-based colorimetric biosensor for the detection of melamine in milk using gold nanoparticles, as presented in Figure 3c, with a detection range from 0 μM to 0.9 μM [29]. Moreover, Figure 3a shows that miRNAs detection has been achieved via a dual-mode sensor based on both colorimetric and fluorescence detection [30]. Furthermore, a colorimetric biosensor based on localized SP resonance via gold nanoparticles has been utilized to detect hydrogen peroxide [31]. However, the localized surface plasmons depend highly on the dimensions, shapes and materials of the nanoparticles, which limits their application. Further, since a large amount of the sample molecules is required for coating the nanoparticle surface, it will not be easy to detect such small molecules. In addition, because of the contamination of the surface in a complex bio medium, such as urine or blood, it makes selectivity much more difficult. Moreover, due to the requirement of lipids and detergents for nanoparticle interference, it is challenging to detect membrane-associated species, which are the major type of drug target.

### 2.2. Optical Coherence Tomography

**Figure 4:**
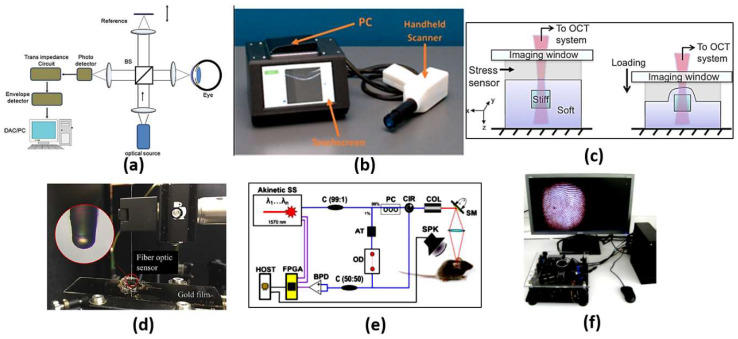
(**a**) Schematic diagram of a typical first-generation free-space optics-based optical coherence tomography setup [32], (**b**) Complete low-cost OCT system with PC, touchscreen and scanner [33], (**c**) Schematics of the optical palpation setup for an inclusion phantom [34], (**d**) Setup of the frequency response measurement of the fiber optic sensor [35], (**e**) Schematic of phase-sensitive OCT [41] and (**f**) A complete compact and mobile FF-OCT fingerprint sensor system, which includes the sensor, the microcomputer and the screen. [42].

Optical coherence tomography (OCT) is an analogous optical technique for ultrasound imaging. The OTC is based on low coherence interferometry to produce cross-sectional images of the tissue. The optical scattering from the tissue is captured, where spatial details of the microstructure are decoded. As may be seen in Figure 4a [32], a super-luminescent diode is used as a light source, where the wave is divided into two parts: sample and reference parts. The first part is scattered from the biological tissue, while the other part is reflected from a reference mirror mounted on the scanning reference optical delay and returns to the detector. At the output of the interferometer, both the reference and sample beams are recombined. The light reflected from the sample and the reference mirror are mixed at a photodetector to form the resultant current signal. Therefore, the OCT system shown in Figure 4a can be used to achieve high-resolution images at different layers, whereby cancerous cells can be distinguished from normal cells based on their disorder and non-homogeneity. In addition, OCT imaging has the advantages of being safe, rapid, non-invasive, and it is recognized as the diagnosis gold standard of retinal-related disease [33]. However, OCT devices are costly and are limited to typical clinical settings. This includes housing in a large tabletop configuration where alternating current power is required, which limits their portability. It is also required from the patient to fix their head on a chinrest without any movement. In addition, a highly skilled technician is often required to align the device, capture the image and perform a quality check to ensure the high quality of the image. Accordingly, a tactile imaging technique for mapping micrometer to millimeter scale mechanical variations in soft tissue has been reported [34]. The OCT system can detect a feature embedded 4.7 mm below the sample surface, as may be seen in Figure 4c. Furthermore, optical coherence photoacoustic microscopy for in vivo imaging has been reported, where a miniaturized Fabry–Pérot interferometer is deposited on a single-mode fiber, as may be seen in Figure 4d [35]. Additionally, OCT can be used for sensing applications, such as monitoring of biochemical-responsive and polymeric micro-particles. In this regard, glucose-responsive micro-particles have been studied by the OCT technique as a proof of concept [36]. Further, an automatic pipeline has been established for tracking the location and size of biochemical-sensitive micro-particles using OCT based on a 3D convolutional neural network (CNN). Such a system has been evaluated in glucose concentration monitoring [37]. In addition, Yi et al. have reported a spectral domain optical coherence tomography, where they succeeded in doubling the imaging depth and imaging speed with no need to increase the spectral resolution [38]. Moreover, remote monitoring has been suggested for biochemical sensing using the distinctive attributes of OCT imaging with biochemical response along with polymeric micro-particles and machine learning [39]. Furthermore, an extended imaging range, reduced sensitivity roll-off and improved light detection efficiency have been achieved by an OCT based on a combined multi-wavelength photoacoustic remote-sensing (PARS) microscope with a swept source [40]. Additionally, a Vernier-tuned distributed Bragg reflector (VT-DBR) swept laser has been used as a source for a fiber-based phase OCT system, as presented in Figure 4e. A phase sensitivity of 0.4 pm standard deviation has been achieved within a factor of less than 2 of the computed shot-noise limit [41]. Further, Figure 4f shows a mobile-based OCT fingerprint biosensor proposed by Auksorius et al. with high accuracy of 74% [42].

### 2.3. Surface-Enhanced Raman Spectroscopy

There are two types of scattering of light by molecules based on elasticity, namely inelastic (Raman scattering) or elastic (Rayleigh scattering). The elastic scattering includes the conservation of photon energy before and after the scattering process. This means that the interaction with the medium molecules is very negligible. Accordingly, very limited information about the medium properties and structure is contained in the elastic scattered light (Rayleigh scattering) [43]. On the other hand, the inelastic scattering may change the photon frequency, whereby a resonance may occur with the vibrational frequency of the molecules of the medium. Accordingly, the excited molecule in the ground state to its first excited vibrational state absorbs the energy lost by the photons (Stokes Raman scattering). Otherwise, a molecule can lose energy in the opposite process, which is absorbed by the photons (anti-Stokes Raman scattering). The photons lost from the medium due to inelastic scattering contain information about the vibrational modes of the molecules. These photons will be absorbed by the incident light. Although Raman scattering is a very weak phenomenon, it is still possible to enlarge the effect via deploying an appropriate nanostructured substrate. The process of enhancing the scattering is concerned with the anti-Stokes process, where the incident photons absorb the energy from the molecules, and hence, the scattered energy is enhanced, which is called a blue shift. It is worth noting that in the Stokes process, the energy is transferred from the incident photons to the molecules, and a red energy shift is obtained. The enhancement of Raman scattering can usually be achieved by deploying a metal colloidal nanoparticle surface, which increases the scattering efficiency [43]. However, there is still a debate on the SERS phenomenon mechanism and the magnitude of the achieved enhancement [43].

**Figure 5:**
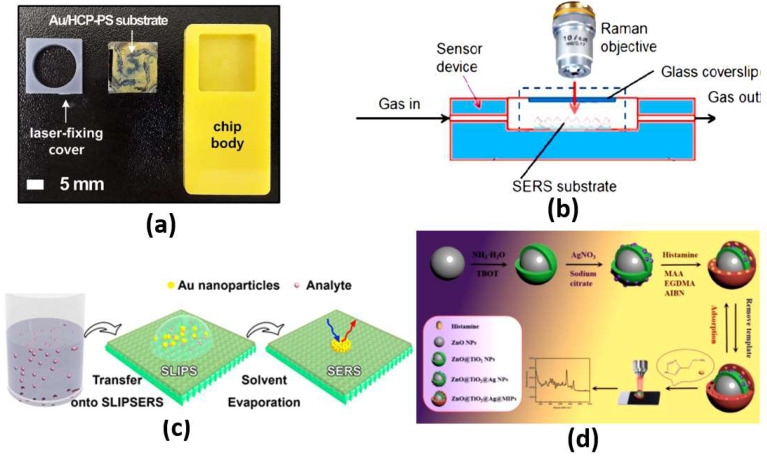
(**a**) Au/HCP-PS monolayer SERS biosensor chip [44], (**b**) Experimental setup for continuous SERS measurements in the gas phase [46], (**c**) Schematic illustration of the liquid phase detection using SLIPSERS [49] and (**d**) SERS sensor for the selective detection of histamine [51].

The most effective scattering methods are chemical and electromagnetic scattering, with large enhancements. In chemical enhancement, a modification in molecular polarizability due to the interaction with the metal can significantly contribute to SERS intensity [43]. Based on the chemical interaction between the noble metal molecules and the probe molecules, chemical enhancement can contribute to only two or three orders of magnitude. On the other hand, by treating the molecules as a point diploe, electromagnetic enhancement drives the molecules to respond to electric fields around the metal surface. The driving electric field is generated from the roughness features that can couple the incident field to surface plasmons. Further, the local field and re-radiation enhancements are available in electromagnetic enhancement. For the local field, a strong spatial localization results from the excitation of surface plasmons, where the amplification of the laser light in small spatial regions is obtained, which is called hot spots. Accordingly, the resulting electromagnetic field in the hot spot regions around the metallic substrate is much higher than that in other regions. In addition, for the re-radiation enhancement case, the Raman power radiated is enhanced due to the presence of the metallic structure nearby the molecule. This is in accordance with the dependency of the power radiated by a dipole on the environment [43]. In this regard, a fabricated plasmonic SERS substrate shown in Figure 5a equipped with a well-aligned, gold-decorated, hexagonally close-packed polystyrene (Au/HCP-PS) nanosphere monolayer has been proposed [44]. Such a plasmonic SERS biosensor provided femtomole-scale detection, giga-scale enhancement and <5% relative standard deviation for reliability and reproducibility, regardless of the measuring site [44]. In addition, non-stoichiometric titanium dioxide and self-assembled quantum-scale-structured (Q-structured) TiO_x_ as an ultra-high sensitive template were employed to intensify the Raman response of tumor biomarkers [45]. The proposed TiO_x_ biosensor achieved a very low limit of detection (LOD) of 1 nM [45]. Moreover, Figure 5b shows a silicon membrane covered with silver-nanoparticles-based SERS, which has been reported for the detection of volatile organic compounds (VOCs) in a gas and liquid environment with a LOD of 1 × $10^{-12}$ M [46]. Furthermore, a SERS liquid sensor for detecting melamine in dairy products has been presented, where only a dilution with double-distilled water and centrifugation is required [47]. With the aid of a silver colloid, a 105-fold enhancement was achieved by the Raman signal for the measurement of melamine, where the reported LOD was 0.01 g m$L^{-1}$ [47]. Additionally, a SERS substrate with a rough gold surface and a specific aptamer has been reported for picomolar detection of ochratoxin A [48]. The proposed SERS sensor is capable of detecting concentrations down to the picomolar range [48]. Moreover, a slippery liquid-infused porous surface-enhanced Raman spectroscopy (SLIPSERS) has been presented and studied with LOD down to ∼75 fM [49]. The reported SLIPSERS is capable of detecting chemicals and biological species, which are either dispersed in liquid or gas phases or bound to solid substrates, as may be seen in Figure 5c [49]. Furthermore, a hydrazine sensor based on modifying 4-mercaptobenzaldehyde (4-MBA) on α-CD-Ag (nanoparticles) NPs was combined with SERS with a corresponding LOD of 38 pM [50]. In addition, a histamine detector has been proposed using a molecularly imprinted SERS sensor [51]. The reported histamine sensor shown in Figure 5d has a linearity over detection range from $10^{-8}\;\mathrm{to}\;10^{-3}\;{\mathrm{mL}}^{-1}$ with a minimum LOD of $3.088\times 10^{-9}{\;\mathrm{mL}}^{-1}$ [51]. Further, an artificial-nose-inspired label-free-arrayed SERS sensor was suggested for fingerprint identification [52]. With up to nine possible combinations, the fingerprint sensor accuracy can approach 100% [52]. However, the EM enhancement is strongly dependent on the localized surface plasmon characteristics. In this regard, the localized surface plasmons are highly dependent on the dimensions, shape and materials of the nanoparticles, which limits their application. Further, a large number of sample molecules are required to coat the nanoparticle surface for the detection of small molecules.

### 2.4. Reflectometric Interference Spectroscopy

Cancer incidence can also be realized through reflectometric interference spectroscopy (RIfS) [53], whereby the target refractive index is determined by variations in the amplitude and phase of polarized light due to the change in the refractive index and thickness of an adsorbed layer of the analyte. Reflectometry can measure the change in optical thickness, which was previously reported as ellipsometry using polarized light. A change in the relative amount of amplitude can be caused by interference at the interfaces of the layer for the two polarized radiation beams. An analytical method has been introduced using the interferometric method called RIfS. This technique is very robust and includes a simple optical detection principle in chemical and biochemical sensing [53]. Based on white light interference at transparent thin layers, the RIfS operates as a label-free optical detection method for surface interactions. In this regard, the light is partially reflected and transmitted at each interface of the thin layers of different materials with negligible absorption. The different beams interfere where the optical path length through these layers is less than the coherence wavelength. The interference pattern depends on the optical thickness, which relies on the physical thickness of the layer, its refractive index, the refractive index of the surrounding medium, the incident angle and the wavelength [53]. The detection principle of the RIfS depends on the change in the optical properties of a given layer system in or at the top layer [54]. A shift of the interference pattern can be caused by the binding of a particle or an analyte molecule to the sensor surface. Moreover, a time-resolved binding curve results from tracking the maximum locus points over time, where the change of the interference spectrum can be used to evaluate the binding signal of the analyte molecule to the sensor surface.

**Figure 6:**
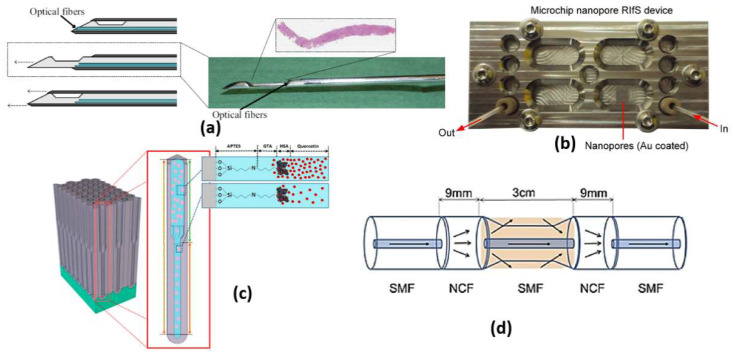
(**a**) Biopsy needle with integrated optical fibers [55], (**b**) Microfluidic nanoporous RIfS device [57], (**c**) Schematic illustration of binding event between human serum albumin (HSA) and quercetin in the environment of fresh primer binding sites (PBS) [60] and (**d**) The structural diagram of the ${\mathrm{Cu}}^{2+}$ sensing part. [61].

The RIfS has a great advantage of its immunity against temperature changes [53], while other refractometric methods, such as ellipsometry and SPR, are highly dependent on the change in the temperature because of the corresponding dependency of the refractive index on temperature. Further, a quick change in the temperature during measurements via these methods is a great challenge. However, in RIfS based on the Clausius Mossotti equation [53], the refractive index n is related to the density, which is in turn related to the thermal expansion. Therefore, with increasing temperature, the refractive index decreases. In addition, a contrary effect arises where thermal expansion of the biopolymer layer causes the physical thickness to increase with the temperature increase. Accordingly, a low impact of temperature change exists on the parameters of RIfS (optical thickness, the product of the refractive index and the physical thickness). In this context, a real-time RIfS based on optical biopsy needle has been introduced by integrating optical fibers at the tip of the needle, as shown in Figure 6a [55]. In addition, sarcosine-imprinted RIfS nanosensor has been reported by using the spin-coating technique [56]. The proposed sarcosine RIfS achieved good linearity, with a correlation coefficient of 0.9622 and a detection limit of 45 nM [56]. Further, Figure 6b presents a label-free RIfS-based microchip biosensor for the detection of circulating tumor cells (CTCs) [57]. Based on the highly ordered nanoporous anodic aluminum oxide, a low detection limit of <1000 cells/mL has been realized for the proposed sensor [57]. The reported mechanism is characterized by high accuracy and a Matthews correlation coefficient (MCC) of 0.93 and 0.87, respectively [57]. Moreover, immuno-sensing for CRP was demonstrated by using the RIfS-based biosensor where an anti-CRP is immobilized using protein A on the SiN chip [58]. Furthermore, a volatile sulfur compound and hydrogen sulfide gas sensor have been suggested based on nanoporous anodic aluminum oxide reflective interferometric sensing [59]. Additionally, a bi-layered nanoporous anodic alumina film with hierarchical funnel-like structure shown in Figure 6c has been proposed and studied [60]. Such a sensor has three layers, which can be used independently for multi-analyte biosensing [60]. Furthermore, a ${\mathrm{Cu}}^{2+}$ ions detection system has been reported based on intermodal interference with temperature calibration [61]. Based on the optical fiber design presented in Figure 6d, the experimental LOD of the reported sensor can reach 3.0 ×$\;10^{-12}$ mol/L with a spectral resolution of 0.02 nm [61]. Moreover, an ethanol sensor has been presented based on the combination of mesoporous silica materials and reflectometric interference technique [44]. The reported ethanol sensor achieved a very small LOD of sub-part per million (<500 part per billion) [62].

It is worth noting that the previous mechanisms are highly dependent on the human senses and require an expert to carry out the analysis, since they are not measurable. Further, a high probability of incorrectness in the diagnoses may arise because of human error. However, other promising mechanisms have been reported and deployed, such as waveguides, surface plasmons and metamaterials. These techniques have measurable quantities with accurate diagnosis. In addition, based on the availability of feasible and accurate measuring instruments, the diagnosis can be performed without the need for an available expert.

### 2.5. Evanescent-Wave-Based Optical Sensors

Evanescent wave is a phenomenon accompanied by total internal reflection (TIR), where part of the wave is leaked through the cladding region in an exponential manner. Based on the non-zero Fresnel coefficient of the transverse electric and transverse magnetic components, upon TIR reflection, an evanescent wave is formed and decays over a distance of nearly 100 nm. In multi-mode waveguides, the refractive indices of the waveguide layers ($n_{\mathrm{waveguide}}$ and $n_{\mathrm{medium}}$), the light incident angle and the wavelength (λ) are the main parameters that affect the penetration depth d_p_ defined as the distance from the surface at which the strength of the field equals to 1/e of its value at the surface. However, the main parameter to control the generation of a considerable evanescent field is the incident angle. The evanescent wave power depends on the incident light on the interface with an angle that is greater than that producing the leaky modes.

#### 2.5.1. Fluorescence Optical Sensors

**Figure 7:**
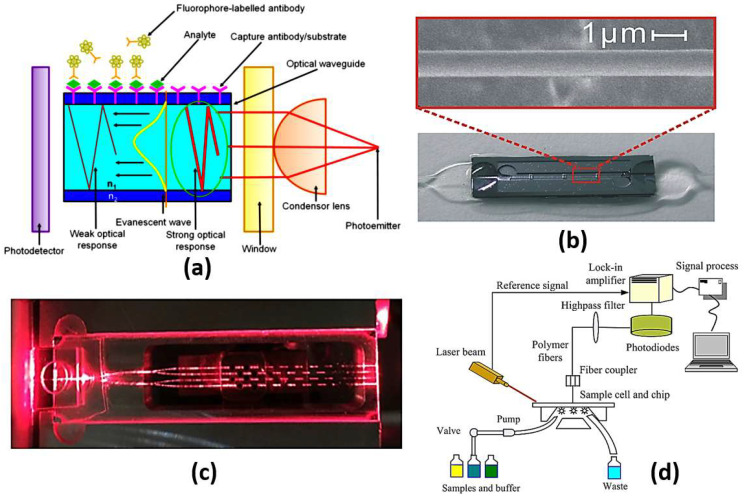
(**a**) A schematic illustration of evanescent-wave-based sensing technique where $n_{2}<n_{1}$ [63], (**b**) Image of a sensor cell and SEM image of a 1.0 mm thick optical microfiber [64], (**c**) A cross-sectional view along one of the sensing patches [65], (**d**) Schematic of planar waveguide evanescent-wave-based immune sensing platform [68].

Due to the surface-sensitive nature of the biosensors based on evanescent wave excitation to generate the fluorescent signal, only the fluorescent molecules near the surface are excited. Based on this limitation, unwanted background signal from a bulk sample can be minimized, while only the signal from fluorophores captured on the surface is enhanced. [63]. Figure 7a shows the schematic diagrams of evanescent-wave-based sensors. As may be seen in Figure 7a, the emitted light signal is concentrated through a condensing lens and enters the waveguide at an angle greater than the critical angle where it undergoes repeated total internal reflections. The region on the outer substrate covered by the analyte under study offers a sufficient matching condition for the evanescent wave to excite the fluorescent signal based on the interaction with the analyte. Finally, the photodetector receives the change in the optical signal from the fluorescent molecules corresponding to the analyte refractive index, as presented in Figure 7a.

What has been most remarkable is the profuse variety of biosensors, which have been developed based on the same fundamental scientific principle. Consequently, the fluorophore receptors on the cladding surface can be excited, and hence, the re-emitted signals can be detected. In addition, the sample to be tested can be added as a medium between the cladding and the fluorophore molecules. Accordingly, it is possible to measure the change in the sample refractive index via detecting the change in the properties of the obtained signal from the fluorophores. Accordingly, an optical microfiber (OMF) biosensor has been demonstrated based on gold nanoparticles (GNPs) as amplification labels for the detection of alpha-fetoprotein (AFP) in serum samples [64]. The detection limits for the proposed biosensor shown in Figure 7b for alpha-fetoprotein are 0.2 ng/mL in PBS and 2 ng/mL in bovine serum, respectively [64]. Further, a 32-analyte integrated optical fluorescence-based multi-channel sensor and its integration with an automated biosensing system have been proposed and analyzed [65]. The multi-channel sensor shown in Figure 7c has a detection limit for aqueous solution of fluorescent dye of 2.8 × $10^{-9}$ M [65]. Moreover, an evanescent-wave-based fluorescence biosensor combined with a DNA bio-barcode assay has been reported and analyzed [66]. Based on magnetic carboxylate particles and non-magnetic carboxylate particles, the suggested biosensor is able to detect a single-nucleotide polymorphism with a detection limit of 2 pM of the target nucleic acids [66]. Moreover, the use of a resonant optical cavity results in an enhancement in the sensitivity of evanescent fluorescence biosensors with reduced sample volumes [67]. Additionally, a planar waveguide evanescent-wave-based immune sensor has been reported for detecting bisphenol A in water samples with a LOD equal to 0.03 mg/L, as may be seen in Figure 7d [68]. Furthermore, a mid-IR optical sensor using the mid-IR fluorescence fibers has been presented for detecting molecules emitted by rare-earth-ion-doped chalcogenide fibers [69]. Moreover, an optical biosensor for detection of bacterial lipopolysaccharide using a red fluorescent protein reporter system has been proposed [70]. The cell-based biosensor was evaluated by testing LPS isolated from 14 bacteria [70].

#### 2.5.2. Plasmonic Biosensors

**Figure 8:**
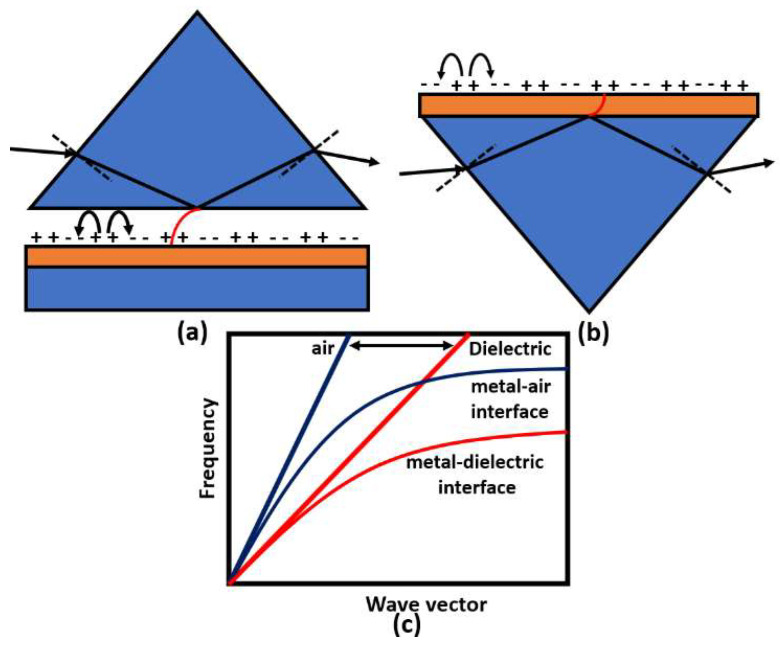
Prism coupling to SPPs based on evanescent wave through total internal reflection using (**a**) Otto and (**b**) Kretschmann configurations. The possible light paths for excitation in the prism coupling and SPP dispersion curve are shown in (**c**).

**Figure 9:**
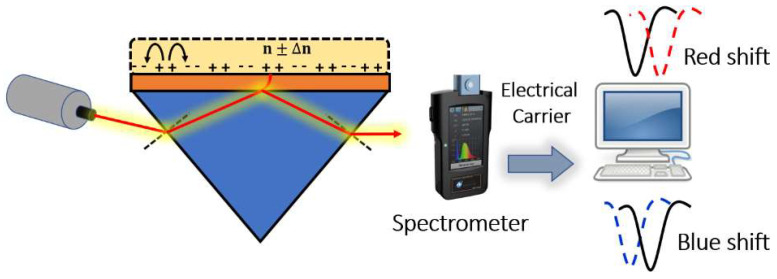
Resonance peaks detected based on prism coupling.

Surface plasmons are electromagnetic oscillations that occur due to the interaction between an incident electromagnetic wave and free electron at the metal/dielectric interface. The idea of plasmonic sensors has been successfully adopted by Otto and Kretschmann [71], where two novel configurations have been reported to excite the surface plasmon polaritons, as may be seen in Figure 8. In both configurations, the incident light beam with a wave vector $k_{0}$ in the air passes through a dielectric prism where the wave transmitted through the medium has a higher value of the wave vector. The new value for the wave vector at the metal–dielectric interface can excite the surface plasmons at the interface. In the Otto configuration, surface plasmons are excited at the outer interface, while in the Kretschmann configuration, the excitation occurs at the inner air–metal interface. It is worth noting that the allowable wave vector values required for excitation lie in the region called the dielectric-air cone, represented in Figure 8c, where $k_{\mathrm{incident}\;\mathrm{wave}}\geq \beta_{\mathrm{surface}\;\mathrm{plasmons}}$. Furthermore, a successful excitation for the surface plasmons is detected as a minimum peak in the reflected beam spectrum. The evanescent wave at the metal/dielectric interface has a greater penetration depth in the dielectric region than that in the metal region. Therefore, the dielectric refractive index has a significant effect on the resonance condition. Based on the mechanism used in the design, the excitation of the surface plasmon wave, and hence, the greatest loss, is observed in the detected spectrum at a specific wavelength or a specific incident angle, as may be seen in Figure 9. Accordingly, by measuring the change in the resonance wavelength or the resonance incident angle, it is possible to measure the change in the refractive index of the sample under test. In this regard, Kabir et al. have proposed an SP Kretschmann-prism-based biosensor for lipid molecules sensing with a corresponding sensitivity of 900 nm/RIU [72]. Moreover, a graphene–MoS_2_ hybrid structure based on the Kretschmann configuration has been proposed [73]. The reported SP sensor with TiO_2_-SiO_2_ nanoparticles has a high sensitivity of 85.375% for formalin detection [73]. In addition, Menon et al. have presented an SP Kretschmann-based biosensor for analyte refractive index sensing with sensitivity up to 540.8°/RIU [74]. The basic idea of plasmonic optical sensors has been extended to other waveguides, including photonic crystal fibers (PCF) and slot waveguides.

#### 2.5.3. Photonic Crystal Fiber Sensors

**Figure 10:**
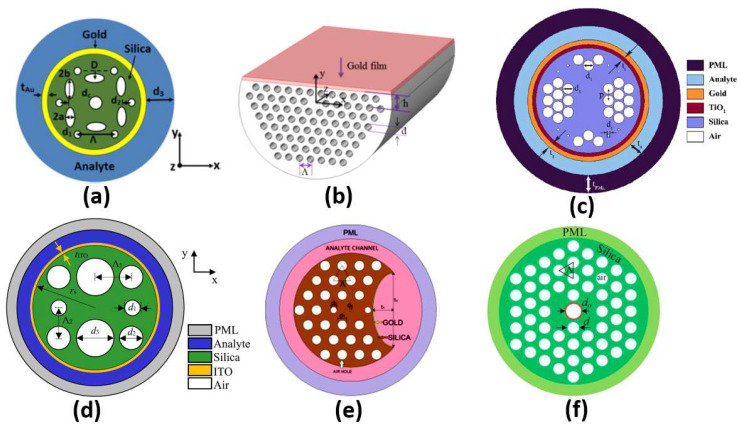
The cross-section of the (**a**) Self-calibration biosensor [80], (**b**) Side-polished D-shaped biosensor [84], (**c**) Ti$O_{2}$ sensor [85], (**d**) ITO-based PCF sensor [86], (**e**) Bended PCF-based SPR sensor [87] and (**f**) Twin-core PCF sensor [90].

Different configurations of plasmonic PCF sensors have been implemented. A coupling occurs between the core guided mode and the plasmonic mode at a resonance wavelength where they have the same effective index. The resonance wavelength depends on the analyte under study. Therefore, a shift occurs in the resonance wavelength when the analyte refractive index changes, which can be used to achieve high sensor sensitivity. In this context, SP resonance (SPR) PCF with sensitivity of 3000 nm/RIU has been reported by Hassani et al. [75]. Additionally, an analyte-filled PCF biosensor with two resonance peaks has been designed with corresponding sensitivities of 2280 nm/RIU and 4354.3 nm/RIU [76]. Moreover, a birefringent plasmonic PCF with supported HEy_11_ HEx_11_ modes offered sensitivities of 2000 nm/RIU and 1700 nm/RIU, respectively [77]. A plasmonic PCF sensor with additional air holes achieved a sensitivity of 4000 nm/RIU [78]. Sensitivities of 1500 nm/RIU and 2000 nm/RIU were achieved by a multi-channel PCF biosensor [79]. Moreover, Hameed et al. have reported a self-calibration SP PCF biosensor, shown in Figure 10a, with sensitivities of 10,000 nm/RIU and 6700 nm/RIU, corresponding to the x-polarized and y-polarized modes, respectively [80]. In addition, a high-wavelength sensitivity of 9000 nm/RIU has been achieved using a gold-coated circular lattice PCF biosensor [81]. Further, an SPR PCF sensor with dual layers of symmetrical square air holes has been reported with sensitivity of 6000 nm/RIU [82]. An ultra-low-loss SPR PCF has been presented for biosensing applications with a wavelength sensitivity of 8500 nm/RIU [83]. Moreover, Wu et al. have reported an SPR biosensor based on D-shaped PCF, as shown in Figure 10b, with a maximum theoretical sensitivity of 21,700 nm/RIU [84]. Further, circular air cavities have been used in an SP PCF biosensor, as shown in Figure 10c, to enhance the performance of refractive index detection with sensitivity up to 41,500 nm/RIU [85]. Additionally, an indium tin oxide (ITO)-coated PCF, shown in Figure 10d, has been presented with sensitivity up to 35,000 nm/RIU for biosensing applications [86]. In addition, Figure 10e presents a bended PCF-based SPR sensor with a gold nanolayer for detecting the refractive index (RI) variations of cancer cells [87]. The reported cancer biosensor is capable of detecting Hela cancerous cells with sensitivity of 7142.86 nm/RIU [87]. Further, a sensitivity of 300 nm/RIU for detection of melanoma cancer tissue has been achieved via nanocavity implementation of SPR biosensors [88]. Moreover, an analytical exploration of a surface-plasmon-resonance- and evanescent-wave-based fiber optic biosensor has been proposed [89] for early detection of breast cancer. Such a biosensor is characterized by a high-amplitude sensitivity of 11,928.25 dB/RIU with a resolution of 8.38 × $10^{-7}$ [89]. Furthermore, a twin-core PCF (TC-PCF) shown in Figure 10f was designed for early detection of blood cancer [90] with high sensitivity of 8571.43 nm/RIU. Moreover, a planner SP multi-mode optical fiber has been demonstrated for refractive index sensing with sensitivity of 608.6 nm/RIU [91]. The reported sensor [91] has been successfully applied for C-reactive protein detection in a buffer solution with a response of 0.118 nm/nM. Furthermore, a multi-functional SP-PCF-based biosensor has been reported and studied [92]. The proposed alcohol-mix-based multi-function sensor is characterized by temperature and refractive index sensitivities of 13.1 nm/°C and 12,700 nm/RIU, respectively [92]. In addition, a multi-analyte sensing capability is provided by a PCF-SPR-based biosensor with four concentric analyte channels [93]. The proposed silver/gold-doped multi-channel biosensor is characterized by sensitivities of 2500 nm/RIU and 3083 nm/RIU for channel 1 and channel 2, respectively [93]. In addition, a heart-shaped dual-core PCF for detecting cancerous cells in human cervical, blood, adrenal glands and breast has been reported with a high sensitivity of 10,000 nm/RIU [94]. Further, a dual-core PCF for the detection of cancer cells in cervical, breast and basal parts has been proposed, as may be seen in Figure 10d [95]. The reported biosensor has a high sensitivity of 10,625 nm/RIU for detecting cervical cancer [95]. However, the PCF sensors reported in Refs [67,68] are based on a coupling between the dual cores without plasmonic materials.

Further, Table 1 presents a comparison corresponding to the application and sensitivity for some of the recent plasmonic biosensors. As may be seen in Table 1 and Figure 10a, adjusting the shape and position of the air holes in the PCF design has a great impact on the resultant sensitivity of 10,000 nm/RIU, as reported in Ref [80], where elliptical shapes have been deployed. Moreover, the D-shaped configuration facilitates the coupling between the core modes and the plasmonic modes near the sensing medium, greatly enhances the sensitivity to 21,700 nm/RIU, as may be seen in Figure 10b and Table 1. In addition, Table 2 and Figure 10c show that doping the SP PCF with a complementary material, such as Ti$O_{2}$, has greatly enhanced the design sensitivity to 41,500 nm/RIU [85]. Furthermore, a PCF with a square lattice has been implemented with ITO to operate in the near-infrared region with an enhanced sensitivity of 35,000 nm/RIU [86], as may be seen in Figure 10d. Although the two designs [87,90] shown in Figure 10e,f have a drawback in terms of sensitivity, they are, on the other hand, novel in the proposed application for early detection of cancer incidence, as presented in Table 1.

#### 2.5.4. Slot Waveguide Sensors

**Figure 11:**
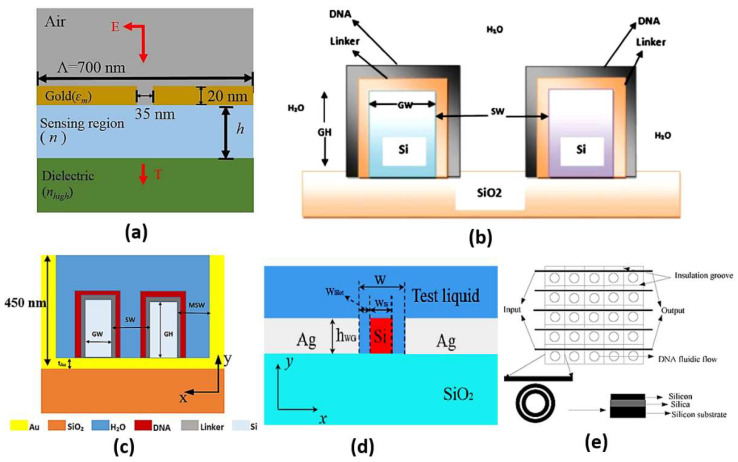
Cross-section of the (**a**) Hybrid plasmonic refractive index sensor with the nano slit array [100], (**b**) Vertical slot waveguide biosensor [96], (**c**) DNA HPSW biosensor [106], (**d**) DSHP waveguide covered with test liquid [110] and (**e**) Compact parallel biosensor [112].

Slot waveguides are based on guiding the light in the hollow or low index region other than the high index region similar to the conventional waveguides. This can be accomplished based on the discontinuity property of the electric field, which allows the confinement of the high-intensity field in the slot region [96]. Accordingly, high light interaction can occur with the studied medium in the slot region, where high sensitivity can be realized. On the other hand, in the conventional waveguides, the confinement of light to the high index region limits the interaction with the sensing medium. Therefore, slot waveguide sensors have attracted high interest. In Ref [97], a dual-slot waveguide based on silicon on insulator (SOI) for refractive index sensing with a sensitivity of 461.327 nm/RIU was proposed. Singh et al. have demonstrated a glucose biosensor using slot waveguide with an average sensitivity of 360 nm/RIU [98]. Additionally, Mere et al. [99] have presented an on-chip chemical biosensor based on a SOI slot waveguide ring resonator with sensitivity of 476 nm/RIU. Further, a numerical study has also been presented on a hybrid plasmonic-mode-based refractive index sensor with sensitivity of 1080 nm/RIU, as may be seen in Figure 11a [100]. Furthermore, a slot waveguide biosensor based on a label-free mechanism for detecting DNA hybridization presented in Figure 11b has been suggested with a sensitivity of 856 nm/RIU [96]. A metal-assisted silicon slot waveguide has been reported for gas sensing, where a sensitivity of 458 nm/RIU has been achieved [101]. Additionally, a sensitivity of 580 nm/RIU has been reported by deploying a hybrid plasmonic-based miniature micro-ring resonator sensor [102]. Further, Kwon proposed a metal-insulator-silicon slot waveguide with a corresponding sensitivity of 430 nm/RIU [103]. In addition, metal coating of dielectric ridges has been applied to a hybrid plasmonic-based slot waveguide for sensing applications [104]. Moreover, a dual polarization measurement in hybrid plasmonic biosensors has been presented and studied [105]. Further, a hybrid plasmonic slot waveguide (HPSW) has been implemented for detecting DNA hybridization, as shown in Figure 11c, with a sensitivity of 1890.4 nm/RIU [106]. Zhang et al. have presented an air-filled gap hybrid plasmonic microcavity for sensing applications with sensitivity of 100 nm/RIU [107]. Furthermore, a vertical slot waveguide has been proposed for biochemical sensing with sensitivity 820 nm/RIU [108]. Xiang and Huang [109] have introduced a slotted photonic crystal waveguide Fano resonant cavity for complex refractive index sensing. The slotted biosensor achieves sensitivities of 494 nm/RIU and ~8850/RIU for real and imaginary refractive index parts, respectively [109]. Moreover, the Mach–Zehnder interferometer shown in Figure 11d was suggested using double slot hybrid plasmonic waveguide for analyte sensing with sensitivity of 1061 nm/RIU [110]. A sensitivity of 893.5 nm/RIU has been achieved using a horizontal slot waveguide biosensor with a ring resonator arrangement [111]. Moreover, a subwavelength grating double slot waveguide presented in Figure 11e has been reported for toxic gas refractive index sensing with a sensitivity of 1000 nm/RIU [112]. Two different configurations based on V-groove silicon on insulator (SOI) waveguides for detection of DNA hybridization have been reported and analyzed [113]. Further, a bandpass optical filter based on a SOI hybrid slot waveguide has been reported for biosensing applications [114]. The reported slot biosensor is characterized by a sensitivity of 270 nm/RIU for on-chip antibody sensing [114]. In addition, an engineered slot waveguide based on Fano resonance has been proposed for detection of viral infections with a sensitivity of 1463 nm/RIU [115]. Moreover, an optical biosensor has been reported based on sidewall grating in a dual slot waveguide modeled on a silicon-on-insulator platform with spectral sensitivity of 661 nm/RIU [116]. Furthermore, a photonic biosensor based on cladding-modulated grating waveguide has been suggested and analyzed [117]. The reported biosensor with three channels on a silicon-on-insulator platform has a sensitivity of 323 nm/RIU [117]. Additionally, an optical slot waveguide with grating-loaded cladding of silicon and titanium dioxide for label-free biosensing has been proposed [118]. The reported biosensor for surface antigen HBsAg detection offers high sensitivity of 1200 nm/RIU [118]. It is worth noting that coupling to the slot waveguide with a photonic strip-slot waveguide is needed, which suffers from undesired back-reflection. Further, mode conversion may occur at the coupling interface, which decreases biosensor sensitivity [119].

A comparison between some recent slot waveguide biosensors is shown in Table 2, including the proposed application and the sensitivity achieved. It can be seen in Table 2 that the sensor’s sensitivity increases considerably by modifying the slot design to increase the light interaction with the sensing medium. For example, a double slot waveguide was used instead of a single slot, where the sensitivity reached a high value of 1000 nm/RIU [100]. Further, the use of a grating structure with a double slot structure increased the sensitivity to 1061 nm/RIU [101]. It can also be seen in Table 2 and Figure 11a that hybrid plasmonic modes can be added to the slot waveguide structure to improve the sensor sensitivity to 1080 nm/RIU. Further, vertical [96] and horizontal designs [111] have been used for the detection of DNA hybridization with sensitivities of 856 nm/RIU and 893.5 nm/RIU, respectively. However, the hybrid plasmonic-mode-based sensors [112] have superiority [96,111] in the sensitivity achieved of 1890.4 nm/RIU, as may be seen in Table 2 and Figure 11c. Further, the metallic grating biosensor [114] has the capability of on-chip biosensing but with a relatively low sensitivity of 270 nm/RIU. Additionally, the modulated cladding reported in Ref [117] has the advantage of on-chip biosensing with enhanced sensitivity of 323 nm/RIU. Moreover, both the engineered slot waveguide [115] and the grading-loaded cladding [118] biosensors achieve relatively high sensitivities of 1463 nm/RIU and 1200 nm/RIU, respectively.

**Table 2:** Comparison between the sensitivities of the recent slot-waveguide-based biosensors.

Design	Application	Sensitivity nm/RIU
Dual-slot waveguide sensor [97]	Lab-on-chip refractive index sensing n~1.326	461.327
Glucose biosensor [98]	Glucose concentration measurement c = 0 mg/dL:200 mg/dL	360
On-chip chemical biosensor [99]	Refractive index sensing n = 1.32:1.34 (Glycerin, potassium chloride and potassium bicarbonate)	476
Hybrid plasmonic mode sensor [100]	Refractive index sensing n = 1.333:1.383	1000
Metal-assisted silicon [101]	Gas refractive index sensing (Acetylene gas) n = 1.000593:1.020593	1061
Miniature micro-ring resonator sensor [102]	Refractive index sensing n~1.33 +1.2e − 4i	458
Metal-insulator-silicon sensor [103]	DNA hybridization detection n = 1.456 (ssDNA): 1.53 (ssDNA)	580
Sensor based on metal coating of dielectric ridges [104]	Refractive index sensing $n_{\mathrm{eff}}=1:1.5$ near 1500 nm	430
Air-filled gap sensor [107]	Refractive index sensing near 1500 nm	1080
Mach–Zehnder-interferometer-based sensor [110]	Aqueous solutions of 2-propanol n = 1.333:1.3776 %C = 0%:100%	494
Vertical slot DNA sensor [96]	DNA hybridization detection n = 1.456 (ssDNA): 1.53 (ssDNA)	856
Horizontal slot DNA sensor [111]	DNA hybridization detection n = 1.456 (ssDNA): 1.53 (ssDNA)	893.5
Subwavelength grating sensor [112]	Toxic gas sensing n = 1.0:1.35 (CO_2_, CH_4_ and CO)	1000
Metallic grating slot waveguide [114]	On-chip antibody biosensing n = 1.32:1.34	270
Engineered slot waveguide [115]	Optical detection of viral infections n = 1.3377:1.3425	1463
Sidewall-grating dual-slot waveguide [116]	Biomaterial sensing n = 1.328:1.338	661
Cladding-modulated grating waveguide [117]	Biomaterial sensing n = 1.33:1.38	323
Grating-loaded cladding slot waveguide [118]	Detection of surface antigen HBsAg n = 1.35:1.39	1200

### 2.6. Metamaterial-Based Sensing

**Figure 12:**
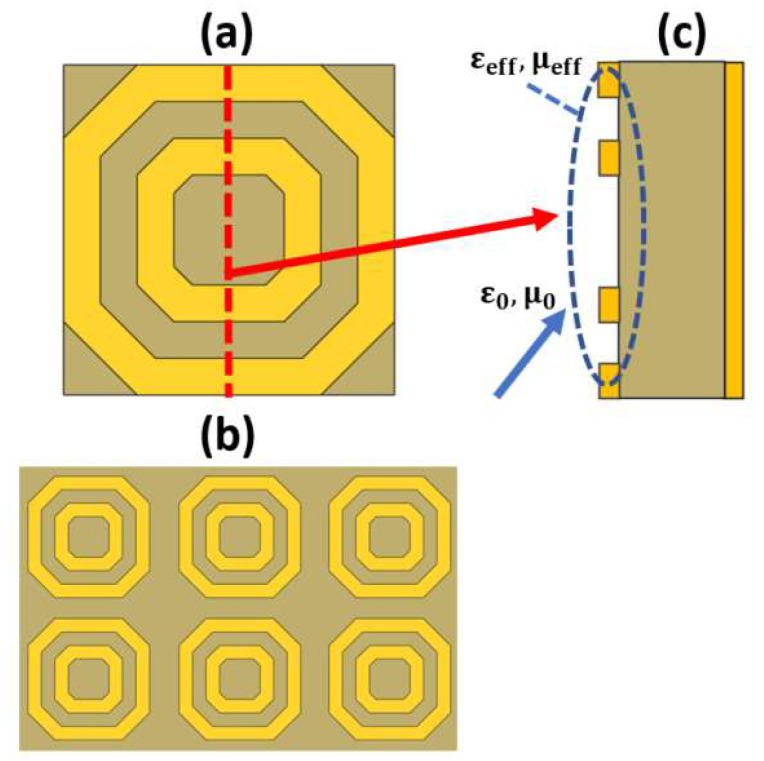
(**a**) Top view of a single-cell split ring resonator [120], (**b**) Top view of the latch array [120] and (**c**) Side view of the sensor to show the effective values for the relative permittivity and permeability for achieving the matching condition.

**Figure 13:**
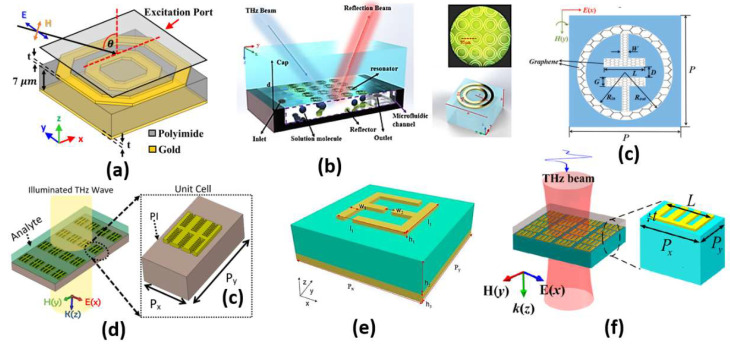
Schematic structures for the (**a**) Hexagonal cancer biosensor [120], (**b**) Microfluidic sensor [125], (**c**) Graphene metamaterial absorber [127], (**d**) Double corrugated metamaterial biosensor [128], (**e**) Unit cell of the F-shaped metamaterial sensor [129] and (**f**) Spoof surface plasmon metamaterials (MMs) structure [131].

Metamaterials are artificially designed structures, which are characterized by properties that cannot be found in natural materials. The most important of these optical properties are negative refractive index and perfect absorption. The design of metamaterials can be achieved on a microscopic scale, which is usually homogenous. In addition, the design can be performed on a macroscopic scale, which is not necessarily homogenous. One of the most famous macroscopic designs is the split ring resonator (SRR), shown in Figure 12 [120], where a dielectric layer is sandwiched between a metallic layer with an arbitrary form and a plane metallic layer with a thickness that is greater than the penetration depth, as may be seen in Figure 12c. The outer metallic layer can be adjusted to take different schematics, as reported in Ref [121]. Therefore, considering an incident electromagnetic wave whose wavelength is greater than the pitch of the metal structure, the wave deals with the structure homogeneously based on the geometrical design. Accordingly, it is possible to control the permittivity and permeability of the overall design via controlling the metal layer structure. The adjustment of the geometrical design can result in an effective impedance, which can be matched to the incident wave impedance. As a result, most of the incident wave passes through the metal design and is reflected back from the other plane metal layer [122]. However, impedance matching is not satisfied from the dielectric side, and the wave is trapped, leading to perfect absorption. As the impedance matching condition is dependent on the properties of the media at the incidence interface, the desired sample can be added at the interface. Consequently, the change in the sample refractive index can be measured through the change in the absorption resonance frequency. In this context, Tao et al. proposed a paper-based metamaterial device for biochemical sensing applications with sensitivity up to 14.3 GHz/mmole $L^{-1}$ [123]. Additionally, two designs for a metamaterial absorber, namely cross-shaped and complementary cross-shaped, have been implemented for analyte refractive index sensing with a sensitivity of 163 GHz/RIU [124]. Furthermore, early detection of cancer has been reported via a metamaterial biosensor [120]. The polyamide-substrate-based hexagonal layers sensor presented in Figure 13a is characterized by high sensitivity in cancer detection of 1649.8 GHz/RIU [120]. In addition, a label-free terahertz biosensor using a metal–air–metal perfect metamaterial absorber has been proposed [125]. The reported sensor shown in Figure 13b [125] with a metallic paired-ring resonator array, a hollow microfluidic channel and a backed reflector achieves normalized sensitivities of 0.51/RIU and 0.47/RIU at 1.28 THz and 0.76 THz, respectively. Further, a biosensor integrated with microfluidics for liver cancer biomarker testing has been proposed, where resonance shifts of 14.2 GHz and 19 GHz have been detected for (0.02524 μg/mL) and (5 mu/mL) corresponding to Gamma-glutamyl transferase (GGT)-II and Alpha-Fetoprotein (AFP), respectively [126]. Moreover, a graphene-based dual-band metamaterial plasmonic perfect absorber shown in Figure 13c has been implemented for refractive index sensing with sensitivity up to 416 GHz/RIU [127]. Furthermore, Niknam et al. have reported a metamaterial terahertz sensor based on a double corrugation form, as presented in Figure 13d, to enhance sensor sensitivity to 1.75 THz/RIU for the refractive index range (1–1.2) [128]. In addition, double-F-shaped metal resonators were deployed in the design of a metamaterial absorber for biosensing applications [129]. The reported biosensor, shown in Figure 13e, achieves a high sensitivity of 1800 GHz/RIU for the analyte refractive index range from 1 to 1.4. [129]. Further, a perfect metamaterial absorber with an absorption ratio of 0.99 has been deployed for layer thickness and refractive index sensing with sensitivities of 23.7 GHz/μm and 300 GHz/RIU, respectively [130]. Recently, Chen and Fan have reported a Fabry–Perot resonance-based planar terahertz metamaterial sensor with sensitivity of 1966 GHz/RIU, as may be seen in Figure 13f [131]. Moreover, Hou et al. have designed and fabricated a bovine serum albumin THz metamaterial sensor, where the achieved sensitivity is equal to 135 GHz/RIU [132]. Furthermore, an all-metal THz metamaterial biosensor has been proposed for protein detection [133]. Such biosensor [133] is characterized by sensitivity to the protein sample of 294.95 GHz/RIU. In addition, a reaction between the carcinoembryonic antigen (CEA) and the anti-CEA has been realized using an antibody-modified metamaterial biosensor with sensitivity of 76.5 GHz/RIU [134]. Moreover, a terahertz metamaterial absorber for sensing biomedical samples has been reported with high sensitivity of 1500 GHz/RIU [135]. Further, a graphene layer was deployed in a folded SRR metamaterial biosensor for tuning the characteristics of the design via an external DC-bias voltage [136]. The reported graphene biosensor had a maximum corresponding sensitivity of 851 GHz/RIU for refractive index values from 1 to 1.6 [136]. In addition, a sickle-shaped metamaterial-based biochemical sensor for detecting chemicals and biomolecules has been reported with a corresponding sensitivity of 502 GHz/RIU [137]. Moreover, a nanorod hyperbolic metamaterial biosensor for biomolecular detection has been demonstrated, whose sensitivity is as high as 41,600 nm/RIU [138]. Furthermore, a bulk refractive index sensor based on 3D composite hyperbolic metamaterial and a graphene film have been deployed in an SP biosensor, where a 4461 nm/RIU sensitivity has been achieved [139]. Additionally, a compact footprint biosensor for cancer detection has been reported and analyzed [140]. Such biosensor has been studied using three types of dielectrics, including silicon dioxide, titanium dioxide and polymethyl methacrylate—substrates with corresponding sensitivity values of 658 nm/RIU, 653 nm/RIU and 633 nm/RIU, respectively [140]. Further, a metamaterial terahertz biosensor with two resonant absorption frequencies has been introduced [141]. Such a biosensor is capable of identification of early-stage cervical cancer tissue with a corresponding sensitivity of 74 GHz/RIU [141]. In addition, a monolayer graphene SPR sensor for aqueous ethanol detection has been reported [142]. The proposed biosensor based on a combination of the virtues of monolayer graphene, hyperbolic metamaterial and D-shaped plastic optic fiber offers a high sensitivity of 5166.7 nm/RIU [142]. Moreover, an asymmetric double-ring resonator metamaterial biosensor has been proposed for detection of biological solutions with sensitivity of 112.05 GHz/RIU [143]. Additionally, a double-U-shaped metamaterial biosensor for label-free and non-destructive sensing of breast cancer cells has been reported with a corresponding sensitivity of 8 GHz/cell [144]. Furthermore, a plasmonic biosensor platform based on hyperbolic metamaterials has been demonstrated for diagnosis of diseases and routine point of care with sensitivity of 30,000 nm/RIU [145]. In addition, a metamaterial biosensor based on arrays of glass substrate coated with single and double Au nanoparticles has been reported for sensing a set of ethanol–water mixtures of different concentrations, where a 320 nm/RIU sensitivity has been achieved [146].

Further, the metamaterial biosensors are based on the famous LC configuration (split ring resonator). According to the pattern and the dimensions of the metallic layers, it is possible to engineer an effective relative permittivity of the structure. The inductance L corresponds to the continuous metallic portions, while the capacitance C is related to the gaps between the metallic layers. Therefore, the incident light wave will interact with the design based on the effective permittivity value seen by the incident light wave. Hence, the resonance frequency and the sensor sensitivity can be controlled.

A comparison between some recent metamaterial designs, including the application and sensor sensitivity, is reported and presented in Table 3. It can be seen in Table 3 and Figure 13 that as the L-C configuration segments increase in the design, the absorption, and hence, the sensitivity to the surrounding medium, can be improved. However, simple L-C configurations with limited segments [125,127] can offer limited sensitivity of around 400 GHz/RIU, as shown in Table 3 and Figure 13b,c. On the other hand, the double ring configuration in Ref [120] based on double hexagonal designs results in an increased number of L-C segments with an enhanced sensitivity of 1649.8 GHz/RIU. Further, the double corrugated design [128] shown in Figure 13d has a different operating frequency of 4 THz rather than the 6 THZ of the F-shaped design reported in Ref [129] (Figure 13e). By controlling the metal layer thickness, the metamaterial design shown in Figure 13f has a high sensitivity of 1966 GHz/RIU, as can be seen in Table 3. In addition, the proposed double hexagonal sensor [120] shown in Figure 13a is novel compared to other designs in the corresponding application of early cancer detection.

Table 4 summarizes the cancer-detection-related biosensors presented in the current review. It can be seen in Table 4 that the proposed sensors in Refs [26,27,28] have considered cancer sensing based on nanoparticle colorimetric detection only where the LAMP assay method is dependent mainly on the experience of the analyst. Further, despite the novelty of the nanocavity sensor [88] in detecting melanoma cancer, the sensitivity achieved of 300 nm/RIU is rather low. On the other hand, the deployment of PCF along with SP has a great impact on the wavelength sensitivity achieved, in the order of 10,000 nm/RIU for cancer detection, as may be seen in Table 4 [87,90,94,95]. Further, despite the novelty of metamaterial sensors [140,141,144] and their application for cancer detection, they are characterized by relatively low sensitivity compared to the other designs, as may be seen in Table 4. Moreover, the detection range (n = 1:2) for the dual resonance peak sensor [141] is greater than most of the other designs suggested, while the compact biosensor [140] has a relatively low precision. In addition, the metamaterial sensor suggested in Ref [126] is characterized by a single loop of metallic layer with single or double gaps, where a sensitivity of only 150 GHz/RIU has been reported. Moreover, utilizing two ring resonators in a metamaterial biosensor [135] increases the sensitivity to 1500 GHz/RIU. Furthermore, different types of cancer cells have been successfully demonstrated for detection using the double hexagonal metamaterial biosensor [120] with a high average sensitivity of 1649.8 GHz/RIU.

The newly adopted optical techniques, such as slot waveguides, surface plasmons and metamaterials, have a high sensitivity compared to the traditional techniques. The new approaches depend on sensing the refractive index variation of the blood samples for early cancer detection. In addition, they do not need an expert to run the test. However, most of the traditional techniques can diagnose cancer at late stages with significant changes in the body. Although the average costs of the new optical systems are comparable to those of the traditional techniques, the operating costs of the new methods are very low; therefore, the overall fees required from the patient can be minimized.

Additionally, a summary of the discussed optical technique platforms’ average costs is presented in Table 5. The table shows that the colorimetric method is the lowest in cost, at USD~800; it requires an expert for operation, and high error degrees may arise [24]. Further, a reflectometric system can cost only USD~1487 and is available from many suppliers [147]. Moreover, a platform based on surface-enhanced Raman spectroscopy phenomena has an average cost of USD~5324 [148]. Furthermore, the optical coherence tomography system suffers from the highest cost among the traditional optical biosensors, with an average cost as high as USD~7164 [33]. On the other hand, the newly adopted optical biosensing techniques have been packaged in platforms for commercial use. In this regard, a slot-waveguide-based biosensing system can be fabricated at an average cost of USD~1000 [149]. In addition, a portable metamaterial-based microfluidic platform has been fabricated at an average cost of USD~4000 [150]. Furthermore, at a cost of USD~6000, a complete surface plasmon analysis system can be purchased from various suppliers [151].

## 3. Future Perspective

The rapid evolution in the medical techniques field has triggered the need for an equivalent evolution in the engineering and technology field. The advancements in medical imaging and radiology led to the development of artificial intelligence (AI) and computer-aided detection (CAD) strategies. Both the AI and CAD approaches are characterized by high accuracy in diagnosis reports and are usually considered as a second opinion for the radiologists. Further, the great enhancement in the AI technology and the capabilities of the CAD systems have promoted them to become high-utility tools [152]. In addition, deep-learning-based AI has a promising role in automated recognition and diagnosis for tasks beyond the human role. In addition, the qualitative potentials of clinicians have been enhanced based on the automated applications of AI. In this context, AI applications, such as tracing numerous lesions at a time, prediction of the resultant tumor by referring to the various databases within a short period, translation of phenotypic variations to genotypic variations and persistent monitoring of patients, have been developed. Further, metamaterial biosensor has been optimized using machine-learning algorithms for the detection of DNA [153]. In order to generate the training data, an iterative transfer matrix approach was used to obtain sets of resonance characteristics in the parameter space for machine learning. It was shown that sensor sensitivity is improved by more than 13 times over conventional techniques using the AI model.

The cancer can also be efficiently classified using an artificial neural network (ANN) [152]. The ANN mathematical model is inspired by the human nervous system with inter-connected neurons. The strategy of the ANN connectionism is designed for computational processing of information. In order to build an adaptive system, the ANN can be used and modified using efficient trained data for a specific application. After the learning process, ANN can be used to detect the performance of the system for untrained input. Further, many networks can be performed simultaneously [152]. As an example, plasmonic sensor has been optimized using the deep-learning method based on ANNs to correlate between the geometrical parameters of the plasmonic sensor and resonance spectra [154]. This will help enhance sensor sensitivity without running any costly simulations. Further, such interesting approach can be adopted for different optical biosensors for early cancer detection with reduced simulation time and improved sensitivity.

It is worth noting that a peer evolution in the physical methods for cancer diagnosis has recently been noticed. In this regard, microwave radiometry has been successfully deployed for diagnosing various urological diseases [155]. The reported method is based on microwave detection of zones with thermal asymmetry, which has advantages of high safety and informativeness over conventional methods, such as X-ray and the ultrasound. Further, these techniques are very helpful for the early stage of inflammatory processing, where standard methods do not provide clear diagnostic solutions [155]. Additionally, the patients’ treatment can be monitored efficiently with different acute pyelonephritis [155].

Inter-patient molecular heterogeneity has also been reported for expanding the variety of anticancer drugs with defined prescriptions [156]. In this context, cancer types with a high degree of intertumoral molecular heterogeneity can theoretically show little response to a specific targeted therapy. Consequently, more types of drugs could be used for clinical use in such cases [156]. Zolotovskaia et al. have reported that the repertoires of current targeted therapeutics do not correspond to the molecular heterogeneities of different types of cancers. However, the recommended cancer drugs are compatible with the gene expression but not gene mutation patterns [156]. Further, lactate-dependent mechanisms of maintaining cancer cell stemness and reparameterization have been studied in Ref [157]. It has been shown that lactic acid is not only a waste product of glycolysis, but it also serves as a signaling molecule [157]. The key role of lactate has been confirmed by the Warburg effect, which is a characteristic of many malignant tumors in physiological conditions and in pathologies [157]. The lactate produces stemness and unlimited cell growth, which can be used by malignant tumors for their initiation and progression [157]. Further, the observed lactatemia in many tumor diseases may be associated with the activation of ancient evolutionary defense mechanisms in order to combat metabolic disorders [157].

## 4. Conclusions

In conclusion, various optical methods for biosensing applications are introduced and reviewed in detail. Different techniques are discussed in terms of the basic idea, detection method and sensitivity value. Optical techniques have been employed in cancer detection, such as colorimetric detection, evanescent wave, surface plasmon resonance and metamaterials. However, it has been shown that traditional methods, such as colorimetric detection, optical coherence tomography, surface-enhanced Raman spectroscopy and reflectometric interference petrography, require an expert technician to monitor the data received. Further, despite the ease of setup and the availability of the equipment required, colorimetric detection depends mainly on the size, shape and material type of the nanoparticles used, which may result in a high degree of fault probability. The same disadvantages are also associated with the SERS technique. Although the OCT is safe, rapid and non-invasive, nevertheless, it suffers from high cost and is mainly used for retinal diagnosis. Further, the RIfS technique is independent of the temperature change. On the other hand, new techniques, such as SP PCF, slot waveguides and metamaterials, depend on measuring the change in resonance wavelength/frequency according to the blood sample under study. Accordingly, such methods are label free, do not require an expert to perform the analysis and can offer the capability of early cancer detection. It is also worth noting that slot biosensors can offer high field confinement in the analyte studied, and hence, high sensitivity can be achieved. Moreover, the SP PCF sensors have high design flexibility and high sensitivity. In addition, metamaterial biosensors can achieve sharp resonance depending on the analyte under study, which can be detected with high accuracy. Further, they are easy to fabricate using the current technology.

## Figures and Tables

**Table 1:** Comparison between the sensitivities of the recent surface-plasmon-based biosensors.

Design	Application	Sensitivity nm/RIU
Enhanced microfluidics sensor [75]	Refractive index sensing n = 1.33:1.34	3000
Analyte-filled core sensor [76]	Refractive index sensing n = 1.46:1.458, n = 1.50:1.52	4354.3
Birefringent PCF SP sensor [77]	Refractive index sensing n = 1.33:1.34	2000
Bimetallic structure sensor [78]	Refractive index sensing n = 1.33:1.34	4000
Multi-channel PCF SP sensor [79]	Refractive index sensing n = 1.33:1.34	2000
Self-calibration SP PCF sensor [80]	Refractive index sensing n = 1.33:1.34	10,000
Gold-coated circular lattice PCF sensor [81]	Refractive index sensing n = 1.34:1.37	9000
Dual-layer SP sensor [82]	Refractive index sensing n = 1.36:1.39	6000
Ultra-low-loss SP sensor [83]	Refractive index sensing n = 1.34:1.37	8500
D-shaped PCF SP sensor [84]	Refractive index sensing n = 1.33:1.34	21,700
Circular-air-cavities-based sensor [85]	Refractive index sensing n = 1.32:1.43	41,500
Indium-tin-oxide-based sensor [86]	Refractive index sensing n = 1.26:1.38	35,000
Hela cancerous cells sensor [87]	Cancer detection n = 1.36:1.395 (Basal), (HELA), (Jurkat), (PC12), (MDA-MB- 231 and MCF-7)	7142.86
Melanoma cancer tissue sensor [88]	Cancer detection (Melanoma tissue)	300
Blood cancer sensor [90]	Cancer detection (Jurkat)	8571.43
Planner SP multi-mode sensor [91]	Biomolecule detection vol% = 0%:30%	608.6
Heart-shaped cancer sensor [94]	Cancer detection (Hela), (Jurkat), (PC12), (MCF-7)	10,000
Cervical, breast and basal parts sensor [95]	Cancer detection n = 1.36:1.399 (Basal), (HELA), (MDA-MB- 231)	10,625

**Table 3:** Comparison between the sensitivities of the recent metamaterial-based biosensors.

Research	Application	Refractive Index Precision	Sensitivity
Double ring sensor [120]	Cancer detection n = 1.36: 1.401	0.001	1649.8 GHz/RIU
Cross- and complementary-cross-shaped sensor [124]	Refractive index sensing n = 1:1.8	0.1	163 GHz/RIU
Graphene-based dual-band sensor [127]	Refractive index sensing n = 1.0:1.5	0.1	416 GHz/RIU
Double corrugation form sensor [128]	Refractive index sensing n = 1.0:1.2	0.2	1750 GHz/RIU
Double-F-shaped sensor [129]	Refractive index sensing n = 1.0:1.4	0.1	1800 GHz/RIU
Perfect absorber sensor [130]	Refractive index sensing n = 1:1.39	0.05	300 GHz/RIU
Fabry–Perot resonance sensor [131]	Refractive index sensing n = 1.0:1.8	0.1	1966 GHz/RIU
Bovine serum albumin sensor [132]	(BSA) detection n = 1.0:2.0	0.2	135 GHz/RIU
All-metal THz [133]	Protein detection n = 1.0:1.4	0.1	294.95 GHz/RIU
Antibody-modified sensor [134]	Carcinoembryonic Antigen n = 1.0:2.0	0.2	76.5 GHz/RIU
Biomedical samples sensor [135]	Cancer detection n = 1.34:1.39	0.05	1500 GHz/RIU
Folded SRR metamaterial sensor [136]	Refractive index sensing n = 1.0:1.6	0.01	851 GHz/RIU
Sickle-shaped metamaterial [137]	Refractive index sensing n = 1.0:1.7	0.1	502 GHz/RIU
Nanorod hyperbolic metamaterial [138]	Biomolecule detection n = 1.3323:1.3329	-	41,600 nm/RIU
3D composite hyperbolic metamaterial [139]	Refractive index sensing n = 1.34:1.352	~0.005	4461 nm/RIU
Compact footprint biosensor [140]	Cancer detection n = 1.35:1.39	0.005	658 nm/RIU
Terahertz biosensor with two resonant absorptions [141]	Cancer detection n = 1.0:2.0	0.2	74 GHz/RIU
Monolayer graphene biosensor [142]	Aqueous ethanol detection n = 1.34:1.352	0.0045	5166.7 nm/RIU
Asymmetric double-ring resonator [143]	Biological solution detection c = 0 Kg/L:0.79 Kg/L	-	112.05 GHz/RIU
Double-U-shaped biosensor [144]	Breast cancer cell detection c = 0 nm/mL:20 ng/mL	-	8 GHz/cell
Hyperbolic metamaterial biosensor [145]	Diagnosis of diseases and routine point of care n = 1.3330:1.3336	0.0006	30,000 nm/RIU
Glass-double Au nanoparticles biosensor [146]	Ethanol–water mixture sensing n = 1.33:1.37	0.001	320 nm/RIU

**Table 4:** Summary of cancer-detection-related research works.

Research	Technique	Measuring Method	Value
Au-Nanoparticle-Decorated sensor [26]	Colorimetric detection	Limit of detection	160 pg/mL
miRNA-155 sensor [27]	Colorimetric detection	Limit of detection	100 aM
Paper-based nanosensors [28]	Colorimetric detection	Limit of detection	$10.0\;\mathrm{ng}\;mL^{-1}$
Hela cancerous cell sensor [87]	SP PCF	Wavelength sensitivity	7142.86 nm/RIU
Melanoma cancer tissue sensor [88]	SP nanocavity	Wavelength sensitivity	300 nm/RIU
Breast cancer sensor [89]	SP optical fiber	Amplitude sensitivity	11,928.25 dB/RIU
Blood cancer sensor [90]	SP PCF	Wavelength sensitivity	8571.43 nm/RIU
Heart-shaped cancer sensor [94]	SP PCF	Wavelength sensitivity	10,000 nm/RIU
Cervical, breast and basal parts sensor [95]	SP PCF	Wavelength sensitivity	10,625 nm/RIU
Double-ring sensor [120]	Metamaterial	Frequency sensitivity	1649.8 GHz/RIU
Liver cancer biomarker sensor [126]	Metamaterial	Frequency sensitivity	150 GHz/RIU
Biomedical sample sensor [135]	Metamaterial	Frequency sensitivity	1500 GHz/RIU
Compact footprint biosensor [140]	Metamaterial	Wavelength sensitivity	658 nm/RIU
Terahertz biosensor with two resonant absorptions [141]	Metamaterial	Frequency sensitivity	74 GHz/RIU
Double-U-shaped biosensor [144]	Metamaterial	Frequency sensitivity	8 GHz/cell

**Table 5:** Average cost of different optical biosensing systems.

Sensor	Cost
Colorimetric [24]	USD~800
OCT [33]	USD~7164
SERS [148]	USD~5324
RIS [147]	USD~1487
SPR [151]	USD~6000
Slot waveguide [149]	USD~1000
Metamaterials [150]	USD~4000

## Data Availability

The data will be available upon request.
